# Person-centered care planning and service engagement: a study protocol for a randomized controlled trial

**DOI:** 10.1186/s13063-015-0715-0

**Published:** 2015-04-22

**Authors:** Victoria Stanhope, Janis Tondora, Larry Davidson, Mimi Choy-Brown, Steven C Marcus

**Affiliations:** Silver School of Social Work, New York University, 1 Washington Square North, New York, NY 10003 USA; Department of Psychiatry, Program for Recovery and Community Health, Yale University School of Medicine, 19 Peck Street, Building One, New Haven, CT 06513 USA; Penn School of Social Policy & Practice, University of Pennsylvania, 3701 Locust Walk, Caster Building, Room C16, Philadelphia, PA 19104-6214 USA

**Keywords:** Implementation, Mental health recovery, Mental health services, Mixed methods, Person-centered care planning, Service engagement

## Abstract

**Background:**

Service disengagement is a pervasive challenge the mental health care system faces. Mental health services are of little value should persons with mental illnesses continue to opt out of receiving them. Consumers attribute disengagement from care to an absence of choice in their treatment. In response, the mental health system is adopting a person-centered model, based upon recovery principles, to engage consumers more actively in their care. Person-centered care planning is a promising practice involving collaboration to develop and implement an actionable plan to assist the person in achieving personal recovery goals.

**Methods/design:**

This study design combines a parallel-group randomized controlled trial of community mental health organizations with qualitative methods to assess the effectiveness of person-centered care planning. Participants at 14 sites in Delaware and Connecticut will be randomized to treatment as usual or the person-centered care planning intervention. Participants will be in leadership (n = 70) or supervisory or direct care (n = 210) roles. The person-centered care planning intervention involves intensive staff training and 12 months of ongoing technical assistance. Quantitative survey data will be collected at baseline, 6 months and 12 months measuringperson-centered care planning competency and organizational factors. Consumer outcomes (engagement, medication adherence, functioning and consumer satisfaction) will be assessed by Medicaid and state-level data. Qualitative data focused on process factors will include staff and consumer interviews and focus groups. In this intent-to-treat analysis, we will use mixed-effects multivariate regression models to evaluate the differential impact of the person-centered care planning intervention on each consumer and implementation outcome as well as the extent to which clinician assessments of organizational factors are associated with the implementation outcome. Mixed methods will triangulate and strengthen the interpretation of outcomes.

**Discussion:**

The aim of this study is to generate valuable guidance for state systems engaged in scale-up and transformation efforts. Targeted staff selection for training to support sustainability will serve to provide further insight into important intervention implementation strategies. Person-centered care planning has the potential to enhance the impact of all evidence-based and recovery-oriented practices and bring practice into line with the emerging national guidelines in health care reform.

**Trial registration:**

This trial was registered with ClinicalTrials.gov (Identifier: NCT02299492) on 21 November 2014 as New York University Protocol Record PCCP-13-9762, Person-Centered Care Planning and Service Engagement.

## Background

Service disengagement is one of the most pervasive and challenging problems currently facing the mental health care system [1,2]. Epidemiological studies have shown that 33% to 55% of people who meet criteria for an axis I diagnosis (according to the *Diagnostic and Statistical Manual of Mental Disorders, Fourth Edition* [3]) have received no mental health care in the past year [4]. Of those who do seek care or are hospitalized, an estimated 24% do not attend treatment sessions as scheduled, 20% drop out of treatment before it is completed, and 18% to 67% do not attend outpatient treatment after hospitalization [1,5]. During the Clinical Antipsychotic Trials of Intervention Effectiveness study, 64% to 82% of patients dropped out of treatment within the first year [6]. No matter how effective mental health services are now or become in the future, they are of little value should persons with mental illnesses continue to choose not to receive them.

Although there are many reasons why people disengage from care [7], people’s experience of the treatment process is one significant factor that can be addressed by practice innovation. Consumers have attributed their disengagement from care to having poor alliances with care providers, including experiences of not being listened to and not being offered the opportunity to make decisions and collaborate in their own treatment [5,8]. In response, the mental health system is moving toward a more person-centered model, based upon recovery principles, to engage consumers more actively in their own care [9]. States have embraced this model in theory, and they are now looking for guidance on how best to implement this model in practice, including how to maximize service quality and consumer outcomes, given the limited resources available to them for workforce development [10]. The proposed study tackles this pressing issue by testing the effectiveness of person-centered care planning (PCCP), a manualized, provider-based intervention that maximizes consumer choice for adults receiving mental health services [11]. By targeting the service-planning process that is shared by all evidence-based practices (EBPs) and mental health services, PCCP embeds a value-added component throughout the agency.

### Person-centered care planning

PCCP makes real a service approach that has been championed by the Institute of Medicine and the New Freedom Commission on Mental Health [12,13]. By shifting from an illness and/or deficit focus to a strengths-based, person-centered one, this intervention fundamentally changes a practice culture that has resulted in many people walking away from the care they need. Instead, PCCP offers an opportunity for people to enter into a genuine partnership with the care system to improve the overall quality and effectiveness of care. PCCP emerged from the federal agenda to transform mental health care to a recovery orientation and is now being adopted at the state level [14]. Models of PCCP were initially developed and found effective for use with persons with developmental disabilities in the 1980s [15,16]. With two randomized trials and evaluations at the state level demonstrating positive results, PCCP has been identified as a promising practice [17,18]. PCCP involves a collaborative process between the service user and all of those people in the person’s life whom he or she identifies as supportive of his or her recovery, including clinical practitioners, other mental health staff involved in the person’s care, and any natural supporters (for example, friends, family members, representatives from faith communities). The aim of the planning process is to develop and implement an actionable plan to assist the person in achieving his or her unique personal goals along the journey of recovery. This plan is also intended to address the specific mental health and/or substance use barriers interfering with the person’s goal achievement, and in this way it is able to meet the rigorous documentation elements required by accrediting and funding bodies (that is, the Joint Commission and Centers for Medicare and Medicaid Services).

Person-centered care plans are (1) oriented toward promoting recovery rather than only minimizing illness and symptoms; (2) based on the person’s own unique life goals and aspirations; (3) focus and build on the person’s capacities, strengths and interests; (4) articulate the person’s own role and the role of both paid practitioners and natural supports in assisting the person to achieve his or her own goals; (5) emphasize the use of natural community settings rather than segregated program settings; and (6) anticipate and allow for uncertainty, setbacks and disagreements as inevitable steps on the path to greater self-determination [10]. These principles make PCCP truly transformative and ultimately sustainable, but they also create challenges when implemented in real-world settings. As with other recovery-oriented practices, PCCP demands a profound shift in values and culture that has implications for providers, organizations and systems [19]. Therefore, sustainable changes in provider behavior may be most likely when training is accompanied by corresponding changes in beliefs, attitudes, and organizational culture, policies and structures.

Prior research has indicated that PCCP is a promising practice across a variety of settings. In one study, researchers randomized five community mental health clinics (CMHCs) to the PCCP condition and five CMHCs to treatment as usual, with providers in the experimental settings receiving training and ongoing coaching in PCCP [18]. The experimental condition had fewer “no shows”, demonstrating higher levels of consumer engagement, and clinicians reported higher rates of medication adherence as compared with the control sites. In a second randomized controlled trial, PCCP was combined with illness management and recovery and a peer-run community integration program for low-income adults of Hispanic and/or African origin with psychotic disorders [17]. The intervention was found to be effective in increasing participants’ active involvement in the care-planning process and in increasing inclusion in the planning process for housing, employment and education. The intervention also increased participants’ sense of control and efficacy in their self-care and overall lives, reduced psychotic symptoms and increased family and social support. Although both of these studies indicated that PCCP was effective, PCCP was combined with other interventions, making its specific impact hard to assess. In addition, researchers have documented concerns specific to implementing recovery-oriented practices, including PCCP. These include beliefs that recovery oriented practice is not unique or novel, that it is too burdensome for overextended clinicians, that it is not desired by people with severe mental illnesses, that it is not evidence-based or reimbursable, that it devalues provider expertise, and that it is too risky because it increases provider exposure to incidents and liability [20,21]. Such concerns pose formidable barriers to implementation if not addressed. To address and further evaluate these concerns, data will be collected on organizational factors to assess if differences in organizational culture impact the implementation of PCCP.

### Conceptual framework and causal model

The primary aim of the study is to examine the effectiveness of PCCP while also seeking to assess the influence of organizational variables on the implementation of PCCP and its impact on consumer outcomes. The conceptual framework integrates implementation science and mental health recovery to hypothesize causal relationships between the organization, provider and service user.

#### Organizational factors

**Figure 1:**
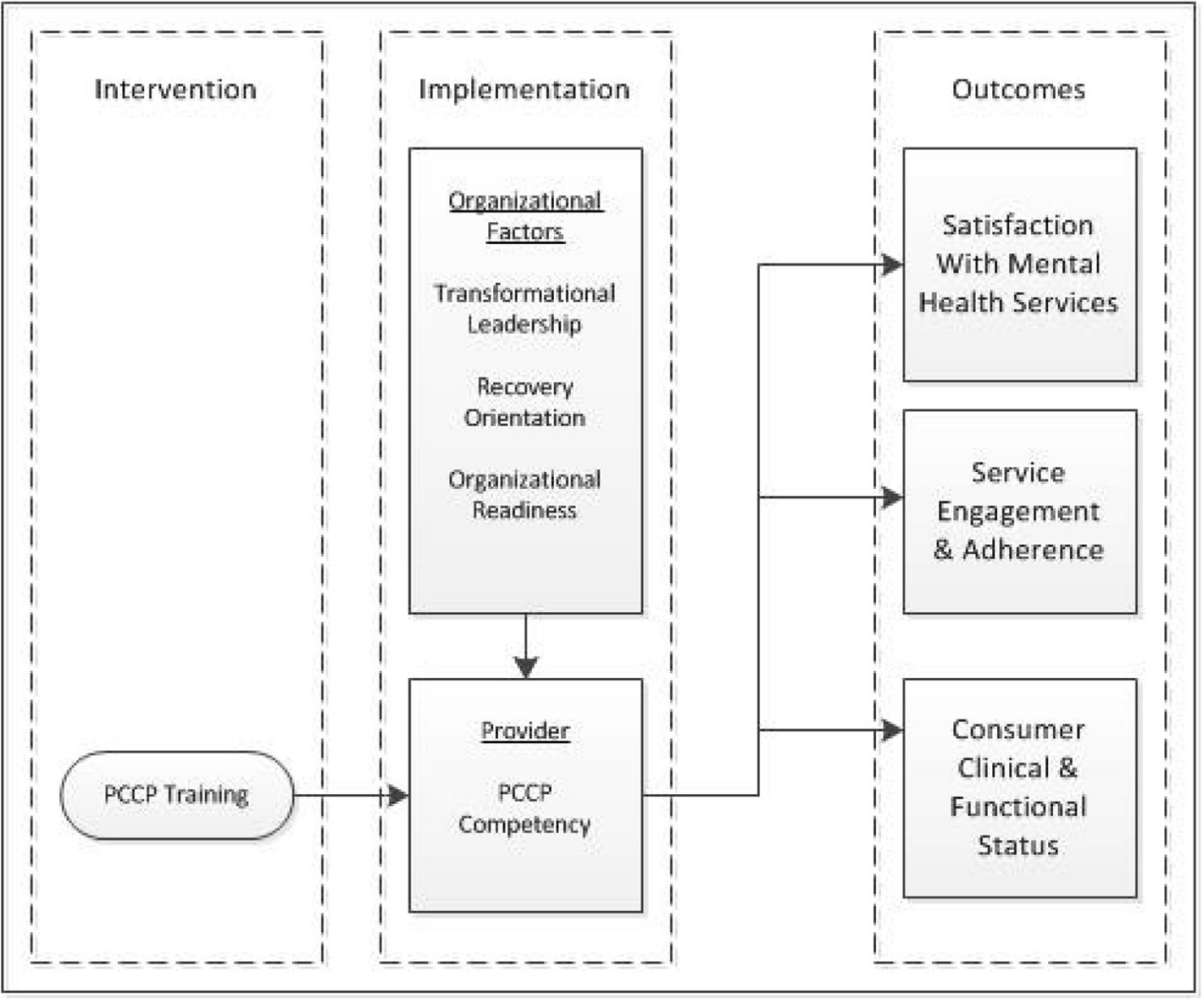
Causal model of person-centered care planning (PCCP).

The organizational level assessment targets three organizational factors: transformational leadership, recovery orientation and organizational readiness. Positive change in these three factors is hypothesized to bring about the necessary organizational conditions for successful training of providers in PCCP and improvement in service user outcomes (see Figure 1 for causal model used in this study).

Transformational leadership is a key upstream organizational factor in creating mental health service organizations that are open to change [22], can improve client outcomes [23] and are recovery-oriented [24]. The leadership qualities that promote transformation are those that inspire providers to adopt the values and goals of their leaders [25]. As opposed to focusing on the completion of specific tasks (transactional leadership), transformational leaders motivate providers by conveying a shared sense of mission and heightening expectations for performance. Recovery orientation is the specific values–innovations fit [26] needed to successfully implement PCCP. Moving toward genuinely person-centered care involves not simply technical skills but also a paradigm shift in how providers understand and value their work with consumers. Nine principles have been identified with regard to recovery orientation, including renewing hope and commitment and redefining self and assuming control [27]. Organizational readiness, which captures both motivation and capacity for change, is now recognized as a critical precursor to successful adoption of innovation in behavioral health care settings [28]. This multifaceted construct reflects how the sum of individual attitudes and behaviors operate at a collective level within an organization [29]. First, staff must be motivated to change by a perceived need for new skills and have confidence in their own ability to adapt to, master and share new skills. Second, organizational climates conducive to change must convey a clear sense of mission and goals, promote staff cohesion and cooperation, and reflect an openness to change. Third, change must be supported by the necessary resources, which include adequate staffing, training, equipment and alignment of procedures [30].

#### Person-centered care planning competency

PCCP competency is the extent to which providers adhere to the intervention (fidelity) and demonstrate the necessary value endorsement and skills to implement PCCP. High-quality PCCP consists of four components: (1) believing people have a right to self-determination, (2) interacting with persons with respect and projecting a hopeful vision for the future, (3) producing a high-quality written service plan and (4) having high expectations for outcomes related to life goals. In accordance with the PCCP manual, the intervention targets provider attitudes and behaviors related to these four areas with training of clinical supervisors and provision of technical assistance (TA) for a 1-year period.

#### Outcomes

Study outcomes are impacted by the choice and perceived relevance of the services offered to consumers. Increasingly, service engagement and adherence are being understood as a function of choice that operates within the broader context of the myriad decisional and social processes that occur when a person enters care [7]. Therefore, whether people choose to use medication or services is determined in large part by the extent to which people feel that they have had a choice leading up to these specific adherence decisions. Self-determination also requires that choices be directly relevant to an individual’s values, preferences and life goals rather than just choosing between limited options that are not perceived to increase quality of life [31]. Service engagement that is precipitated by an increase in self-determination has been found to be associated with greater satisfaction with services [32]. PCCP seeks to maximize choice and ownership of the treatment process by engaging people in care that is relevant and responsive to their holistic needs and life goals. This increases the likelihood that they will adhere to and benefit from treatment. Making decisions, considered a fundamental “capability” needed in order to pursue a meaningful life [33], nevertheless may not be a readily accessible skill for those who have not had control over their lives for many years. PCCP also addresses what Deegan referred to as “learned helplessness” [34] by offering consumers not only choices but also the supports and skills they need in order to achieve higher functional status and regain a sense of control over their lives.

## Methods

This trial has three aims. Aim 1 and Aim 2 utilize quantitative methods and Aim 3 utilizes qualitative methods.

Aim 1 is to assess the effectiveness of PCCP. Aim 2 is to assess organizational factors in the implementation and effectiveness of PCCP.

### Participants

We randomly selected 14 research sites from among all community mental health clinics (CMHCs) in Delaware and Connecticut. These agencies serve approximately 8,000 consumers. We used the Power and Precision software package (Biostat, Englewood, NJ, USA) to calculate sample sizes needed to achieve 80% power (α = 0.05) to detect these effects in our two-level hierarchical design where clinicians and patients are nested within CMHCs. The software accounts for this nesting using the intracluster correlation coefficient (ICC), a measure of the relatedness of clustered data that compares the variance within and between clusters. The ICC in our pilot data was 0.013. To achieve the study aims, we will need 280 participants (20 per site). We will need 1,125 medical record reviews (75 per site).

The services delivered at the CMHCs include psychiatric crisis intervention, individual and group therapy, psychiatric evaluation, medication management, case management, community support programs and rehabilitation services. A total of 280 providers will be recruited for the study. Within each research site, five clinical supervisors, ten direct care staff and five leadership staff will be recruited to participate in the study. The supervisors are clinical staff who are directly responsible for supervising the direct care staff. Direct care staff are clinicians who provide frontline services to consumers without any supervisory duties. The supervisors and direct care staff work in the outpatient mental health programs listed above. They come from a variety of disciplines, including social work, psychology, psychiatric rehabilitation, counseling and peer support. The leadership staff are in administrative positions and do not directly supervise direct care staff. Their titles include executive director, medical director, administration direction, operations director, quality improvement director, clinical services directors, peer support or consumer affairs director, training and staff development director, rehabilitation services director, human resources director, and representatives from the agency’s consumer advisory council.

Each supervisor will nominate two direct care staff who report to them. They will nominate individuals based on whether they fulfill the criteria of change agents, which are that they have worked at the agency for more than 1 year, have demonstrated leadership potential, are open to change and adoption of new practices and are perceived as a resource and/or role model by other direct care staff.

Secondary data will be used to assess consumer outcomes. Consumers eligible for this study will be adults who have received services at the CMHCs continuously for 1 year prior to the study. We will use the Medicaid and state mental health data of all eligible consumers.

### Randomization

The 14 sites (8 in Connecticut and 6 in Delaware) selected for the study will be randomized into the 2 study arms, yielding 7 sites per condition, balanced by state. A research team member will use SAS 9.3 software to generate the allocation sequence assigning sites to condition. The software will generate randomized lists blocked by state, ensuring three experimental sites and three control sites in Delaware and four experimental sites and four control sites in Connecticut. The responsible team member will be blinded to site-specific information during randomization. Blinding is not possible, given the circumstances of the intervention.

### Intervention

PCCP provides a framework for the collaborative cocreation of a recovery-oriented treatment plan that is driven by an individual’s most valued life goals. Providers will learn how to elicit and empathize with their client’s subjective experiences as a whole person and how to help the consumer identify and articulate interests, preferences and personal recovery goals. Challenges include reframing symptoms and impairments as barriers to goal attainment; reframing the use of medications as tools for overcoming these barriers and moving ahead in one’s life; instilling hope and encouraging the person’s incremental efforts in the face of fear, uncertainty and demoralization; identifying short-term, realistic and measurable objectives that can be achieved within the plan period of 3 to 6 months while keeping these objectives explicitly connected to longer-term aspirations that might span years; and expanding a person’s network to include natural supporters as well as professional care providers.

The PCCP implementation strategy targets change in direct service provider behavior by providing training and follow-up TA to clinical supervisors and nominated direct care staff over a yearlong period. This targeted staff selection is aimed at contributing strategies for scaling up and sustaining practices in large mental health systems. This strategy serves to integrate ongoing, knowledgeable supervision of direct care staff from onset. Additionally, the nominated direct care staff are selected in part for their demonstrated leadership potential. Clinical supervisors and direct care staff will employ this interpersonal influence and formal and informal leadership roles as “change agents” within their teams and programs [35,36].

To ensure rigorous testing, this implementation-as-usual strategy will follow the current “gold standard” for training in EBP. This includes a workshop with behavioral rehearsal (that is, practice), ongoing supervision and coaching on skills, and a provider training manual to reinforce learning [37,38]. Clinical supervisors and direct care staff will receive 2-day training aimed at providing practical, nuts-and-bolts guidance for how to maintain a strengths-based recovery orientation within a comprehensive, person-centered plan that also meets rigorous documentation standards. The training will address how to provide PCCP and how to transfer this knowledge and skills via supervision to direct services staff. It begins with a brief introduction to the broader construct of recovery-oriented care to ensure that participants have a shared conceptual foundation for the more advanced PCCP skills training. The content of PCCP training is organized around a published logic model [11] that walks supervisors through a series of steps in creating person-centered recovery plans. These steps include initial request for services, comprehensive strengths-based assessment, shared prioritization of action areas, collaborative development of recovery plan content (including goals, strengths and/or barriers, and short-term objectives), delivery of services consistent with an established plan, and monitoring of both the quality of the planning process and its impact on consumer outcomes. In each of these areas, opportunities to maximize the person-centered nature of both the process (that is, how roles, relationships and planning meetings look different in PCCP) and the documentation (that is, the resultant written recovery plan) are highlighted. Significant emphasis is placed on the changing roles of both the consumer and the practitioner in PCCP, with tools provided to support the development of competence and confidence in these shifting roles.

Trainees will be given the provider training manual as well as a consumer toolkit*.* The provider manual is a published 154-page guide developed by the Yale Program for Recovery and Community Health. The consumer toolkit, also developed by Yale Program for Recovery and Community Health, is a 24-page guide designed to explain to consumers the process of person-centered care planning. Designed for administrators and providers, the manual gives detailed step-by-step guidance on how to write person-centered plans for people receiving behavioral health plans. To strengthen the transfer of learning from the initial training to participants’ daily work, clinical supervisors will be asked to participate in two follow-up TA telephone calls per month. The first call of each month will be a case presentation and consultation call. The supervisor’s team will be invited to participate in the call, and training consultants will offer live modeling of how supervisors should conduct these PCCP feedback sessions with their supervisees. The second monthly call will involve the clinical supervisors only and will be fully dedicated to supporting supervisors with implementation issues they encounter as they encourage the adoption of PCCP by their team members. The training team will keep logs of the training and record process notes of TA calls to measure the frequency and intensity of training given to each provider.

### Measurement

The intervention will be conducted over a period of 12 months and will be staggered across experimental sites. Organizational factors and PCCP competency will be measured by surveys of the leaders, clinical supervisors and direct care staff because reliable assessment requires multiple perspectives [30]. These surveys will be administered at baseline, 6 months and 12 months. Chart reviews will also be used to measure PCCP competency. Service engagement and consumer outcomes will be measured using state mental health data and Medicaid claims data.

Online assessment measures will be administered to all study participants, regardless of treatment condition. Researchers will collaborate with each CMHC to identify the leadership staff, clinical supervisors and direct care staff. An e-mail with an embedded URL linking participants to the online consent form and survey will be sent to each provider individually. Depending on their role in the agency, staff will receive one of the three surveys for direct care staff, clinical supervisors or executive leadership. The surveys are substantively equal with variation in the measurement versions and included language. Surveys will be completed at three time points: baseline, the midpoint of the intervention period (6 months) and postintervention (12 months). Each research site in the control condition will be paired with a site in the experimental condition, and the surveys will be e-mailed to pairs 1 month prior to the experimental site’s receipt of the PCCP intervention. Chart reviews will assess 75 service plans per site before and after the intervention.

#### Person-centered care planning competency

##### Person-Centered Care Questionnaire, administrative and provider versions

The Person-Centered Care Questionnaire (PCCQ), created in 2009 by Tondora and Miller of the Yale Program for Recovery and Community Health, is a 32-item measure designed to assess the degree to which service planning is person-centered, with higher scores associated with a more person-centered service plan. This measure has not been validated. The PCCQ has been used to measure intervention fidelity by the investigative team in their work with multiple states. Respondents rate either their service-planning processes or the staff processes on a 5-point scale ranging from “strongly disagree” to “strongly agree”.

##### Person-centered care planning Documentation Quality Review Tool

This tool lists ten key indicators of person-centered care values and principles that can be applied to clinical record documentation. The measure generates a score from 0 to 10 (yes-or-no answers to ten indicators), with a higher score reflecting more person-centeredness. The PCCP Documentation Quality Review Tool was developed by the investigators in collaboration with the New York Association of Psychiatric Rehabilitation Services. This is not a validated measure, but an initial pilot administration of the tool in one large New York CMHC was positively received by the agency administration, both for its breadth of content and for its ease of administration.

#### Organizational factors

##### Multifactor Leadership Questionnaire, leader and rater (staff) versions

The Multifactor Leadership Questionnaire 5x-Short Form [39] is a widely used measure of leadership in organizations, and its dimensions have been associated with organizational performance and success [22]. This study focused on transformational leadership, which is composed of five subscales: idealized influence (belief of leadership character; α = 0.91), idealized influence (observed leadership behavior; α = 0.86), inspirational motivation (α = 0.94), intellectual stimulation (α = 0.93) and individual consideration (α = 0.87) [25]. Clinical supervisory and direct care staff were asked to rate the executive leadership, and the executive leadership were asked to rate themselves, on a 5-point frequency scale ranging from “not at all” to “frequently, if not always”, with higher ratings indicating a higher degree of idealized influence, inspiration, intellectual stimulation and individual consideration.

##### Organizational Readiness for Change Scale, director and staff versions

The Organizational Readiness for Change Scale [30] is used to assess provider perceptions of the readiness for change. This self-administered measure has four domains: motivational factors, staff attributes, program resources and organizational climate. This study focuses on the 30-item organizational climate domain with six subscales that correspond to organizational features associated with implementation of innovative practices [40,41]. The selected subscales include mission, cohesion, autonomy, communication, stress and change. Each subscale has acceptable internal consistency values ranging from 0.76 to 0.82 [30]. Respondents are asked to rate the working environment at their agency on a 5-point scale ranging from “strongly disagree” to “strongly agree”, with higher ratings indicating higher degrees of climate for change.

##### Recovery Self-Assessment–Revised, administrator and provider versions

The Recovery Self-Assessment–Revised scale (RSA) [42] is a 36-item measure assessing the degree to which recovery-oriented practices are implemented in an agency. Respondents are asked to rate the agency as a whole on a 5-point scale ranging from “strongly disagree” to “strongly agree” with items related to the scale’s five components: life goals, involvement, diversity of treatment options, choice and individually tailored services. Higher scores indicate a higher integration of recovery-oriented practices. The RSA has been used as a quality improvement and/or research tool in over 35 states and in several different countries. In previous research, the RSA has been demonstrated to have excellent internal consistency, reliability and validity [42-44].

#### Consumer outcomes

##### Community mental health visits

Mental health visits at each agency are reported via the CMHC Service Ticket Form, which is completed by providers after each scheduled appointment or unscheduled visit. Visits are coded as no show, canceled, unscheduled or scheduled.

##### Outpatient mental health service use

Medicaid claims data will be used to measure use of medication management, case management, individual and group psychotherapy services, and rehabilitative services. Relevant claims will be identified based on presence of an International Classification of Diseases, Ninth Revision, Clinical Modification (ICD-9-CM), mental health diagnostic code as primary diagnoses on evaluation and management visit claims or by corresponding Current Procedural Terminology (CPT) codes in all outpatient settings except emergency departments.

##### Medication adherence

Medicaid claims data will be used to measure adherence to psychiatric medications. We will calculate adherence to newly initiated and ongoing use of antidepressants, antipsychotics and mood stabilizers using the medication possession ratio (MPR) [45]. The MPR is a continuous measure of the usable days supplied from all prescriptions in each period divided by the number of days in each period.

##### Psychiatric hospitalizations and emergency department use

Medicaid claims data will be used to measure psychiatric hospitalizations and emergency department use identified by relevant ICD-9-CM diagnostic codes (290 through 314), CPT codes, place of service and revenue center codes. The occurrence of any psychiatric hospitalization, the number of hospitalizations and the length of stay for each will be coded.

##### Employment, housing and forensic involvement status

This information will be extracted from the state Annual Consumer Reporting Form, which contains questions about (1) primary employment, secondary employment and hours worked per week during the past 90 days; (2) residence and whether the consumer has been homeless in the past 30 days; and (3) current legal involvement.

##### Consumer satisfaction with services, social connectedness and improved functioning

These domains will be measured by using state data from the Consumer/Client Satisfaction Survey, which consists of the state version of the Mental Health Statistics Improvement Program Consumer Survey, version 1.2 [46]. This 43-item measure has 23 items that measure consumer satisfaction with services, 13 items that measure functioning, and 4 items that measure social connectedness. Higher scores reflect greater satisfaction, functioning and connectedness.

#### Consumer demographic and clinical characteristics

Consumer diagnosis, comorbid medical and substance abuse diagnoses, sex, age and race will be obtained from the Medicaid claims data for inclusion as covariates in our models.

### Quantitative Data analysis

All analyses will be done by intent to treat (ITT). Differences across the two arms of the study (PCCP and treatment as usual) will be analyzed using the PROC MIXED statement in SAS statistical software (SAS Institute, Cary, NC, USA) to create mixed-effects multivariate regression models. Mixed-effects models will permit the appropriate analysis of nesting of study subjects within sites and repeated measurement of outcomes for subjects over time. Descriptive statistics will be examined for treatment groups and for additional groups as needed (for example, treatment completers versus dropouts). In addition, individual and average trajectories will be plotted for all repeated measures over time according to assigned treatment.

Bivariate analyses will be conducted using *χ*^2^ tests for categorical variables and *t*-tests for continuous dependent variables at each time point to evaluate the differential impact of the PCCP intervention on each consumer and implementation outcome. All models will control for, as fixed effects, baseline consumer factors as appropriate (diagnosis, comorbid medical and substance abuse diagnoses, sex, age, race and psychiatric hospitalization rates). Models will be conducted separately for outcomes from leadership staff, clinical supervisors, chart reviews, state mental health data and Medicaid claims. The unit of observation in all models will be, as appropriate, clinical supervisors, organizational leaders or consumers.

In the analyses for aim 1, we will assess the effectiveness of PCCP on service engagement and clinical outcomes. We will use each outcome as the dependent variable and a dichotomized measure of the PCCP intervention (any intervention versus no intervention) as our independent variable. Controlling for relevant baseline characteristics and including a fixed effect for state, we will use separate mixed linear regression models to determine the impact of the intervention on each of the outcomes at each time point. The primary ITT test and estimation will be done at the 24-month time point with tests at earlier time points. We will then conduct a growth curve analysis with repeated observations representing measures at baseline, 12 months and 24 months. The analysis will include variables for time, treatment and their interaction, but it will be focused on the magnitude and statistical significance of the time × treatment interaction to quantify the relative difference in the rate of outcome change between the treatment groups. All models will include random intercepts and slopes for CMHC and state. We will repeat these analyses to compare each intervention arm to treatment as usual.

The analyses for aim 2 are limited to only clinicians and consumers in agencies exposed to the intervention. Within this population, we will use linear mixed-effects regression models to determine the extent to which clinician assessments of organizational factors, including transformational leadership, recovery orientation and organizational readiness (independent variables), are associated with our primary implementation outcome, which is clinician PCCP competency (dependent variable). Competency will be measured as change from baseline to 12 and 24 months and examined separately at each time point. Our first model will include one observation per clinician and will include a random intercept for organization, but no independent variables. This will allow us to calculate the maximum proportion of variance attributable to the organization (that is, the ICC). A second model will also include our three independent variables as fixed effects. This model will allow us to examine the individual contribution of each organizational factor to explaining competency and also to understand how much of the total explainable organizational variation in our dependent variable from the first model is explained by the three organizational factors in this model. In subsequent models, we will use these same techniques to assess the impact of organizational factors on patient outcomes, including consumer satisfaction with services, service engagement and adherence and consumer clinical and functional status.

Aim 3 is to use qualitative methods to understand how care planning impacts service engagement and how implementation processes influence organization and provider level behavior.

### Participants

The seven experimental research sites will be selected for qualitative inquiry. Leadership staff, clinical supervisors participating in the training, their direct care staff, and consumers receiving services from them will be recruited for the study. Researchers will contact CMHC staff to explain the study and identify providers and consumers for recruitment. Flyers describing the study to providers and consumers will be distributed to each CMHC to explain the study and give a toll-free telephone number with which potential participants can contact researchers directly.

### Procedures

The qualitative component of study will take place at the conclusion of the 12-month intervention period for each of the experimental sites. Interviews and focus groups will be conducted to understand staff perspectives on new practices and to identify barriers to and facilitators of implementation [28,47]. Guided key informant interviews will be conducted with five leadership staff members from each site. We will also conduct three focus groups in each agency: one with clinical supervisors, one with direct care staff and one with consumers. We will recruit 8 to 12 participants per group. The interviews and focus groups with staff will be guided by a review of the RSA and PCCQ–provider version scores obtained at baseline and 6 months. Staff will be asked about service planning and its influence on engagement and consumer outcomes. The consumer focus group will be guided by the Person-Centered Care Planning Quality Indicator, which has seven items about consumers’ experience of service planning. Consumers will then be asked about their engagement in services and how this relates to the service-planning process. The research team will coordinate with the CMHC director or designee to schedule interviews and focus group meetings.

### Qualitative data analysis

Data will be analyzed according to grounded theory, one of the most systematized of the qualitative methods [48]. Grounded theory encourages inductive thinking and use of the constant comparative method [49]. Our goal is to gain an in-depth understanding of the barriers to, facilitators of and consumer and provider perceptions of service planning, implementation of PCCP and how service planning relates to service engagement and consumer outcomes. Focus group transcripts will be analyzed for themes and divergent opinions with commonly used qualitative data analytic procedures [50]. These methods will include a component of independent review by more than one person, a discussion and convergence of themes and consensus development. To ensure a maximum degree of trustworthiness and minimize bias, we will deploy strategies for rigor, which will include team debriefings, negative case analysis and audit trail.

### Mixed methods

The purpose of mixing methods in this study is to use qualitative data to (1) triangulate and expand on the quantitative data on implementation outcomes and (2) interpret the quantitative data on PCCP outcomes. First, focus groups and key informant interviews will triangulate the findings from the implementation outcome measures by allowing us to examine whether participants perceived the implementation strategies to result in the successful uptake of PCCP. Triangulation enhances rigor by providing more than one source of data to examine whether findings are congruent [51]. The qualitative data can compensate for the potential lack of power in this study to conduct hypothesis testing on implementation outcome variables. The qualitative data will also provide more in-depth data on the implementation process, particularly on unanticipated facilitators and barriers. Second, the focus groups and key informant interviews will generate data on the effectiveness of the PCCP intervention, giving us insight into why PCCP is effective or is not, and they will explain potential variation across sites in PCCP outcomes.

### Ethical issues

For the quantitative component of the study, potential risks are minimal for providers participating in the intervention and completing the surveys. The intervention involves training and consultation that are part of ongoing quality improvement activities instigated by the state. Informed consent will take place prior to participants’ completing surveys. We will initially send the providers a notification e-mail, briefly describing how the study is an examination of PCCP and inviting their participation. If they choose to continue, they will be presented with the informed consent statement form to complete. The online survey does not ask for sensitive information, but subjects will be able to refuse to answer any survey question. Confidentiality will be protected for both the individual respondents and the CMHCs; CMHCs will not have access to employees’ responses.

For the qualitative component of the study, potential risks to participants are minimal. Informed consent will be obtained prior to the interviews and focus groups. The content of the interviews and focus groups will be confidential and will not be reported to CMHCs. The study has been approved by the New York University Institutional Review Board (number 13–9762). These methods adhere to the Consolidated Standards of Reporting Trials guidelines.

## Discussion

The study has a real-world setting within agencies that are subject to changes based on funding and policy changes. Given that the duration of this study is 5 years, we anticipate that there may be structural changes in some of these settings that may impact the implementation of the study. There also may be agency-specific initiatives and implementation of new practices as a result of transformation efforts, particularly in relation to health care reform, that may affect the PCCP process. A potential limitation of the study is that the transfer to PCCP competency from clinical supervisors to direct care staff will not be directly measured. Therefore, the lack of effect could potentially lie in the implementation strategy of training supervisors rather than in the PCCP intervention. However, the qualitative inquiry, which will be focused on the experimental groups, is designed to gain an understanding of the implementation process, and, through interviews of both direct care staff and supervisors, data related to the transfer of PCCP competency will be collected. Another limitation of the study is that consumer outcomes will be measured only with secondary data gathered for Medicaid purposes and state-level reporting by agencies. Therefore, we will not be able to report on more nuanced outcomes related to relationships with providers and subjective mental health recovery.

## Conclusions

Designed to bridge the science to services gap, this study is focused on how agencies can bring about the wholesale transformation needed to deliver sustainable person-centered care. The PCCP intervention is designed to address a pressing public health problem—the high rates of disengagement from public mental health services. The aim is to generate valuable guidance on how state systems engaged in the transformation process can best use their limited resources during times of significant fiscal constraint. The study has support from both state policy makers and individual agencies, meaning that that the findings will likely inform future policies and practice. PCCP has the potential to enhance the impact of all EBPs and recovery-oriented practices and bring practice in line with national guidelines that have emerged as a result of health care reform.

By focusing on training supervisors in addition to direct care staff, the study can increase its external validity and build CMHC internal capacity. Training supervisors based upon our experience of conducting statewide training is the most efficient and feasible way to promote agency-wide transformation. We can considerably extend the reach of PCCP with this strategy, allowing for large-scale replication implemented at the state level. Also, training supervisors increases the sustainability of PCCP by building internal skills and capacity among a more stable clinical leadership group, as opposed to focusing efforts on a direct service provider group, which tends to suffer from high rates of turnover within public sector mental health systems. The study addresses implementation by measuring organizational factors to assess both whether the intervention is effective and under what conditions it is effective. In addition, in the qualitative inquiry, we will examine the implementation process from the perspective of multiple stakeholders. By paying attention to organizational factors, we aim to inform the next step in PCCP research, which is to design and test implementation strategies to ensure the successful adoption of PCCP in real-world settings.

## Trial status

Recruitment, baseline data collection and training of experimental site personnel have been initiated at the research sites. Recruitment of staff began in October 2014.

## Abbreviations

CMHC: Community mental health clinic
CPT: Current Procedural Terminology code
EBP: Evidence-Based Practice
ICC: Intracluster correlation coefficient
ICD-9-CM: International Classification of Diseases, Ninth Revision, Clinical Modification
ITT: Intent to treat
MPR: Medication possession ratio
PCCP: Person-centered care planning
PCCQ: Person-Centered Care Questionnaire
RSA: Recovery Self-Assessment–Revised
TA: Technical Assistance
